# Myosins, an Underestimated Player in the Infectious Cycle of Pathogenic Bacteria

**DOI:** 10.3390/ijms22020615

**Published:** 2021-01-09

**Authors:** Margaux Pillon, Patricia Doublet

**Affiliations:** 1 CIRI, Centre International de Recherche en Infectiologie, Legionella Pathogenesis Group, Université de Lyon, 69007 Lyon, France; margaux.pillon@univ-lyon1.fr; 2 Institut National de la Santé et de la Recherche Médicale (INSERM), U1111, 69007 Lyon, France; 3 Ecole Normale Supérieure de Lyon, 69007 Lyon, France; 4 Centre International de Recherche en Infectiologie, Université Claude Bernard Lyon 1, 69007 Lyon, France; 5 Centre National de la Recherche Scientifique, UMR5308, 69007 Lyon, France

**Keywords:** myosins, bacteria, infectious cycle, hijacking, cellular pathways, host-pathogen interactions

## Abstract

Myosins play a key role in many cellular processes such as cell migration, adhesion, intracellular trafficking and internalization processes, making them ideal targets for bacteria. Through selected examples, such as enteropathogenic *E. coli* (EPEC), *Neisseria*, *Salmonella*, *Shigella*, *Listeria* or *Chlamydia*, this review aims to illustrate how bacteria target and hijack host cell myosins in order to adhere to the cell, to enter the cell by triggering their internalization, to evade from the cytosolic autonomous cell defense, to promote the biogenesis of intracellular replicative niche, to disseminate in tissues by cell-to-cell spreading, to exit out the host cell, and also to evade from macrophage phagocytosis. It highlights the diversity and sophistication of the strategy evolved by bacteria to manipulate one of their privileged targets, the actin cytoskeleton.

## 1. Introduction

The eukaryotic cell cytoskeleton is a complex and dynamic network that shapes the cell and plays a key role in numerous cellular processes such as cell migration, adhesion, internalization and intracellular trafficking. To achieve all these dynamic events, the cell cytoskeleton involves several proteins, among which the subject of this Special Issue, the myosins.

Myosins constitute a superfamily of actin-based motor proteins that share properties of actin-binding, chemical-to-mechanical energy conversion (ATPase activity) and directed movement along actin filaments (generally to the plus-end filament) [1]. All myosins are composed of three domains, namely the head, neck, and tail domains, forming the myosin heavy chain. Localized at the N-terminus, the globular head domain contains evolutionarily conserved actin- and ATP-binding sites with ATPase activity. This domain is thus dedicated to the motor function of myosins by generating force via the ATP hydrolysis and by converting chemical energy into mechanical work [2,3]. The central neck domain, also known as the “lever-arm”, is an α-helical segment [4] that amplifies the movement originating from the motor domain. Following ATP hydrolysis, the lever-arm region rotates to amplify the angstrom-level conformational changes in the motor domain to the nanometer-sized power-stroke motions [5]. It contains one or several IQ motifs whose function is to bind regulatory elements of motor function, such as calmodulin or myosin light chains (MLC) [6,7]. Finally, the C-terminal tail domain, as the most variable region of the myosins, determines the specific cellular functions of the different myosin classes by presenting different binding sites to molecular cargos such as SRC Homology 3 (SH3), GTPase-activating protein (GAP), four-point-one, ezrin, radixin, moesin (FERM) or Pleckstrin homology (PH) domains [8]. For some classes, such as myosins II and V, the tail region may also contain regions predicted to be coiled-coil domains when myosins molecules form dimers.

Human myosins, classified in around 18 families [9], participate in a diversity of cellular processes, including cell migration and adhesion, signal transduction, intracellular and membrane trafficking [10,11]. In addition to these essential functions for the eukaryotic cell, it recently appeared that some myosins, from class I, II, VI, VII, IX and X, are also at the heart of the infectious cycle of many intracellular bacteria. Specifically, given the key role of myosins in eukaryotic cell biology, bacteria have evolved strategies that target and hijack the host cell myosins to their own benefit. After a brief reminder of important insights on the structure and function of myosins of interest, this review aims at summarizing how some bacterial pathogens associated with important human diseases, such as EPEC, *Neisseria*, *Salmonella*, *Shigella*, *Listeria*, and *Chlamydia*, target host cell myosins to adhere to the host cell, to enter into the host cell, to evade autophagy/inflammasome degradation, to exit from the host cell, or to evade phagocytosis, ultimately promoting successful infection.

## 2. Structural and Functional Insights on Myosins I, II, VI, VII, IX and X

### 2.1. Myosins I

**Figure 1:**
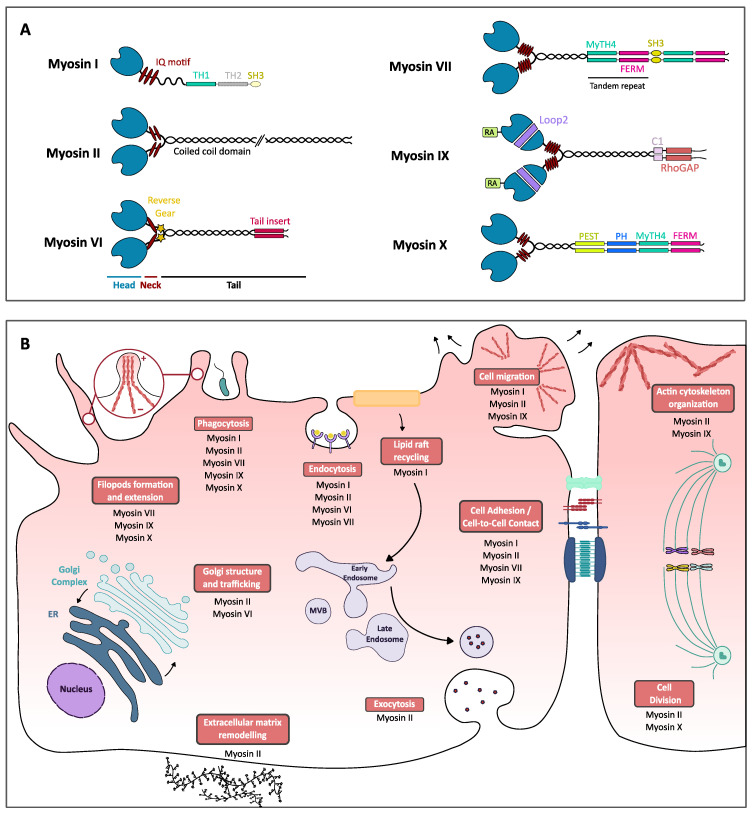
Myosins structure and functions in eukaryotic cells. (**A**) Schematic structure of different classes of myosins. Myosins are all composed of three distinct parts, the head, neck and tail regions. The head (in blue) is the most conserved region as it contains the motor domain as well as the ATP- and actin-binding domain. Class IX myosin head is preceded by a unique extension, an Ras-associated (RA) domain. The neck can vary in the composition in IQ motifs (red ellipses) able to bind different regulating molecules such as the myosin light chains or calmodulin. In the class IV myosin neck region, the stars represent an insertion called the reverse gear that permits the myosin VI to walk in the opposite direction as other myosins. The tail (in black and colored boxes), the most variable domain, is the region where coiled-coil domains can be found, ensuring myosins dimerization, as well as other specific domains that can bind different cargos, which can be related to myosins’ cellular functions: myosin tail homology 4 (MyTH4) domain-binding cytoskeletal proteins such as microtubules, four-point-one, ezrin, radixin, moesin (FERM) domain, mostly found in cytoskeletal-associated proteins, that allows proteins to bind membrane proteins, SH3 (Src homology 3) domain-binding to adaptor proteins or phosphorylated tyrosine residues, C1 or phorbol esters/diacylglycerol-binding domain, Rho GAP-domain-binding Rho GTPases, PEST region found on proteins with short half-life and Pleckstrin homology (PH) domain, allows proteins to bind to phosphoinositids and therefore to the plasma membrane [1]. Class I myosins can present short-tail (continuous line) or long-tail isoforms (discontinuous line) thanks to supplementary domains. (**B**) Schematic representation of cellular processes (boxed in red) and the associated myosins. This representation is not exhaustive of all the roles of myosins.

Class I myosins act as a monomer of one heavy chain with (i) a conserved motor domain, (ii) a lever-arm domain called light chain-binding domain (LCBD) that binds one to six calmodulin-like light myosin chains or calmodulin itself, and (iii) the tail domain that contains a tail homology 1 (TH1) domain with a high-affinity for anionic phospholipids [12,13]. Depending on the presence and number of other additional domains named TH2 and TH3, the eight isoforms of class I myosins (Myo1A to Myo1H) are sorted into two different classes based on the length of the tail domain (Figure 1A).

Because the tail domain binds phospholipids, myosin I is the bridge between plasma membranes and the actin cytoskeleton, which gives it a key role in cellular adhesion and plasma membrane deformation [14]. Class I myosin also regulates the actin cytoskeleton, as exemplified by Myo1c that interacts with G-actin through its C-terminal cargo-binding tail domain and transports G-actin to the leading edge of the cell for facilitating actin polymerization involved in cell migration [15]. In addition, class I myosin would be involved in endocytosis and intracellular trafficking, as Myo1e depletion in HeLa and SK-MEL-28 cells reduces transferrin endocytosis and trafficking [16]. Importantly, Myo1g plays an important role in FCγR phagocytosis as it presents the ability to bind both F-actin and membrane phospholipids, thus facilitating contraction of the phagocytic cup and resulting in the cup closure [17]. Finally, Myo1c is involved in the recycling of lipid rafts that impact cell spreading as well as their migration [18] (Figure 1B).

### 2.2. Myosins II

Class II myosins are subdivided into two categories, muscular and non-muscle. Only non-muscle class II myosin will be discussed in this review. Class II non-muscle myosins isoforms NMIIA, NMIIB and NMIIC are encoded by three genes (MYH9, MYH10 and MYH14) that are expressed ubiquitously in human cells. Class II myosin structure follows the myosins classic structure composed of the head, lever-arm and tail domains, but the uniqueness of myosin II lies in its ability to create bipolar filaments [19]. Indeed, after dimerization and upon phosphorylation of its two regulating myosin light chains [3,6], myosin II homodimer conformation changes, which allows interaction through its C-terminal tail with another myosin II homodimer to form a bipolar filament (Figure 1A). These filaments display different structures and lifecycle dynamics, depending on their isoform composition. Given class myosin II isoforms are characterized by specific cellular location and function [20,21], these filaments are thus involved in many mechanical tasks, including the regulation of the actin cytoskeleton, organization and cellular life cycle events such as adhesion, migration, cytokinesis, or extracellular matrix remodeling. More recently, non-muscle myosin II-activated (phosphorylated on myosin light chains and unfolded) monomers have been shown to be involved in intracellular trafficking (endocytosis, exocytosis, phagocytosis and vesicular transport) (Figure 1B). For instance, class II myosin is required for clathrin-mediated endocytosis [22], plays a role in the Golgi to ER transport [23] and contributes to complement receptor (CR)-dependent phagocytosis via a Rho/ROK/myosin II pathway [24] that results in membrane ruffles formation to capture particles that are opsonized with C3b complement [25].

### 2.3. Myosins VI

Class VI myosin is one of a kind as it is the first actin-based motor discovered, presenting the ability to move along actin filaments in the opposite direction of other myosins [26]. Its unique ability to move towards actin minus-end is due to the presence of a 53-residues insert, the insert-2 in the neck/lever arm region, which induces a 120° curvature of the helix alpha sufficient to invert Myo6 directional “walking” (Figure 1A). Insert-2 is thus also called the “reverse gear” [27,28]. Class VI myosins also contain differential sequences in their tail domain depending on alternative splicing in specific tissues. Thus, the human Myo6 sequence displays in its C-terminal domain a 31-amino acid large insert, a 9-amino acid small insert or no insert at all, defining a large isoform or a small isoform (small insert or no insert) [29].

Depending on its isoform type, class VI myosins take part in several cellular processes, among those, clathrin-mediated endocytosis, Golgi structure and trafficking as well as cellular migration. The large Myo6 isoform can directly bind clathrin, thanks to a specific α2-linker region, and Dab2, a clathrin adaptor protein, through its C-terminal domain [29], thus proposing that Myo6 may be directly involved in the transport of endocytic receptor to relocate it to clathrin-rich regions when bound to the ligand. Noteworthy, class VI myosin’s unique ability to “walk” along actin filaments towards the minus-end could support this hypothesis as microvilli are composed of actin filaments bundles polarized with the plus-end on top of microvilli [30]. The second role of Myo6 in endocytosis would be the formation of clathrin-coated vesicles, as suggested by the direct interaction between large Myo6 isoform and clathrin light chain a (CLCa), one component of clathrin three-legged molecule that plays a regulatory part on assembly, disassembly and rigidity of clathrin cage [31]. Class VI myosins could also play a role in late endocytosis to ensure the transport of uncoated vesicles through the rich-actin regions in the periphery of the endocytosis site to the early endosome [32]. Class VI myosins have also been proposed to play a role in the Golgi structure. Class VI myosins localize at Golgi complex, associates with trans-Golgi network (TGN), and it would create a bond between Golgi membranes and actin filaments to maintain the complex reticular structure of Golgi [33,34]. Myosin VI could also play a role in transporting cargo vesicles on short distances along actin filaments from the TGN to microtubules that are used for long-range transport. As myosin VI participates in the formation of endocytic clathrin-coated pits, it can be proposed that it would support TGN vesicles formation and interfere with protein secretion, as shown by the 40% decrease of reporter-protein secretion in Snell’s water cells when myosin VI is mutated [33](Figure 1B).

### 2.4. Myosins VII

The class VII isoforms myosin Myo5a and Myo5b contain a head domain containing actin- and ATP-binding sites, a neck domain with 5 IQ motifs and a tail region with a short coiled-coil region similar to those of myosin V and X, which stabilizes a dimeric structure [35]. Importantly, the tail region also contains two large tandem repeats separated by an SH3 domain, each composed of a β-integrin-binding FERM domain and a microtubules-binding MyTH4 domain [36].

The C-terminal domain of myosin VII interacts with vezatin, a transmembrane protein that binds β-catenin/E-cadherin complex involved in cell adhesion and cell–cell junctions. This connection between myosin VII and adhesion complex would be involved in the myosin VII defect found in Usher syndrome type 1 (USH1B) [37,38]. Nevertheless, the main role of myosin VII regarding host–pathogen interactions is to facilitate phagocytosis by extension of filopods, specifically by mediating surface adhesion to particles at the tip of filopods in the early stages of phagocytosis. Thus, in *D. discoideum*, myosin VII would form with talin A, a dynamic adhesion complex at the cell membrane, stabilized at the phagocytic cup by vacuolin, that would facilitate the phagocytic extension and uptake of particles [37,38,39] (Figure 1B).

### 2.5. Myosins IX

Two isoforms Myo9a, Myo9b and their several splicing variants are part of the class IX myosins in mammals. In addition to sharing the common structure of myosins, i.e., head, neck and tail domains, myosin IX contains an N-terminal extension as well as a large insertion composed of basic residues in the loop2 of the head domain. Although the extension presents no known binding partner, the insertion in loop2 has been found to interact with actin filaments and displays a calmodulin-binding site [40,41]. In addition, the myosin IX tail domain contains a functional RhoGTPase activating protein (RhoGAP) domain that switches small GTPases, preferentially targeting RhoA GTPase, from an active ATP-bound state to an inactive ADP-bound state [42] (Figure 1A).

Class IX myosins have been defined as motorized signaling molecules consistently with their role in the organization of actin, in regulating cell–cell contact and more broadly, cellular differentiation and migration. Noteworthy, instead of “walking” on actin filaments, the Myo9b motor domain promotes gliding towards the plus-end of actin filaments like an “inchworm” [43,44,45]. In addition, the ATP-bound form of myosin IX presents a high-affinity for actin, whereas other myosins are not associated with actin in this state [46,47]. This ATP-form high-affinity for actin is suggested to be the consequence of the large insertion in loop2 in the head domain and its interaction with actin filaments [44]. Given its RhoGAP domain, myosin IX is expected to be involved in Rho-dependent processes, such as the formation of actin stress fibers, of focal adhesions attaching cells to a surface, of lamellipodia and filopodia-dependent cellular adhesion and migration [48,49]. Consistently, Myo9b was found to accumulate at polymerizing actin filaments sites such as extending lamellipodia or at the extremity of filopodia [48]. Despite that the molecular mechanism is not yet identified, Myo9a was proposed to play a role in cell–cell contacts by regulating RhoGTPases during the collective migration of 16HBE cells, a key process for tissue morphogenesis [49] (Figure 1B).

### 2.6. Myosins X

Myosin X, widely expressed in vertebrate tissues, follows the same structure as other myosins, with a head domain, a neck domain, and a tail comprising a dimer-forming coiled-coil motif [50] and multiple cargo-binding domains related to myosin X functions. Indeed, the tail region contains 3 Pleckstrin homology (PH) domains implicated in PI3K signaling, microtubules-binding MyTH4 domain and β-integrin-binding FERM domain [51] (Figure 1A).

Early on, myosin X was found to be enriched in filopods, involved in adhesion, signaling, and environmental sensing, and to move along actin filaments present in filopodia in both directions [52]: a “forward” movement as myosin X moves toward the plus-end of actin filaments and thus the filopodial tip, consequence of its motor activity; or “rearward” movement as the myosin X goes back to the cellular body. Thanks to its intrafilopodial motility, myosin X, mostly in its dimeric form, takes part in filopodial formation initiation [52,53], elongation [54], as well as inducing integrin relocalization [55]. Myosin X is also involved in the mitotic division to maintain spindle orientation by the mean of integrin-mediated cellular adhesion [56]. Finally, the myosin X second Pleckstrin Homology (PH) domain binds PIP3, which promotes its recruitment to phagocytic cups necessary for optimal FCγR-mediated phagocytosis in macrophages [57].

## 3. Myosins and Their Role in Pathogenic Bacteria Infectious Cycle

Due to their central role in eukaryotic cell biology, myosins have been shown to be targeted by bacteria to reach certain stages of their infectious cycle, as revealed by the impact of myosin inhibition and/or silencing on bacterial adhesion, entry, spread or evasion from immune defenses. Although the molecular mechanism leading to myosin hijacking is not always clearly established, it relies either (i) on the direct interaction of one or more bacterial effectors—i.e., bacterial proteins injected inside the host cell by bacterial secretion systems such as the Type 3 secretion system (T3SS)—with myosin, (ii) on the direct interaction of bacterial effectors with a positive or negative regulator of myosin, such as MLCK or MYPT1, or (iii) on the triggering by bacterial effectors or host–cell contact of complex signaling pathways leading to the activation or inhibition of myosin.

### 3.1. Myosin I—Targeting by Enteropathogenic E. coli (EPEC) to Contribute to Adhesion and to Evade Macrophages Phagocytosis

**Figure 2:**
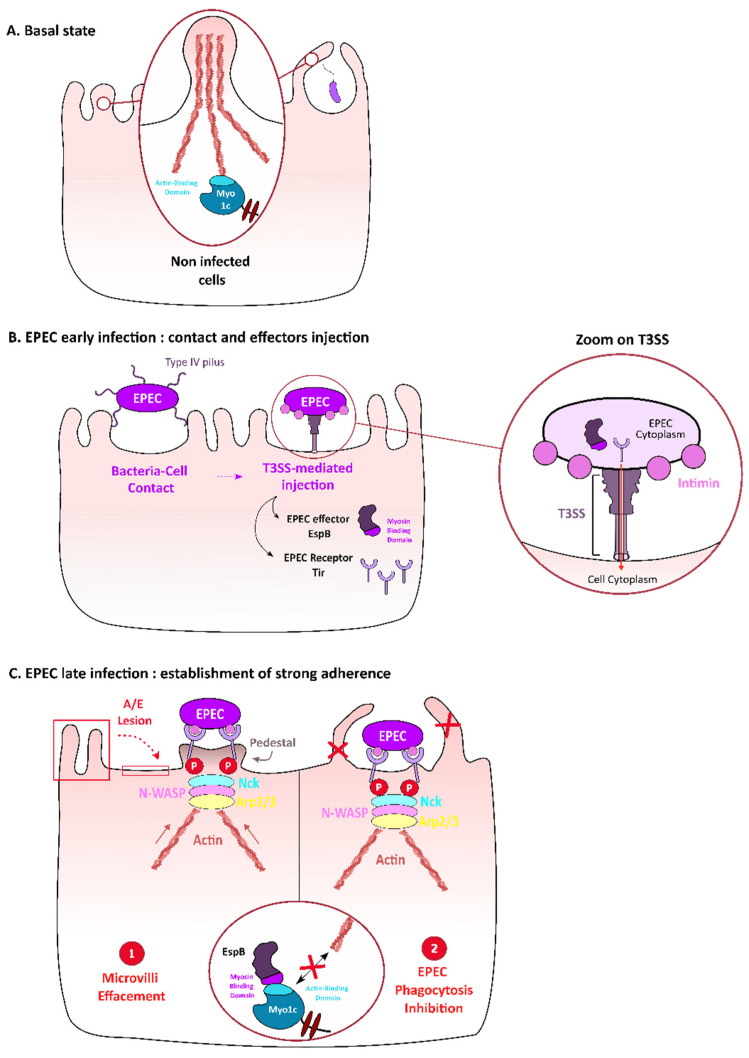
Role of Myo1c in enteropathogenic *E. coli* (EPEC) adhesion on intestinal epithelial cells surface and in evasion from phagocytosis. Microvilli and phagocytic extensions rely on the actin cytoskeleton, in particular on myosin Ic that interacts with actin (**A**). When EPEC infects intestinal epithelial cells, the bacterial Tir receptor is injected by EPEC’s *Salmonella typhimurium* Type 3 secretion system (T3SS) and then presented at the cell host surface to interact with the EPEC envelop protein intimin. T3SS is a molecular device that allows the injection of bacterial proteins inside the host cell cytoplasm (zoom) (**B**). This interaction triggers actin polymerization under the bacterial adhesion site through activation of the Nck/N-WASP/Arp2/3 pathway, thus leading to the formation of a platform called “pedestal” (C1). The effector EspB also injected inside the host cell by the T3SS (**B**), competes with actin for myosin-binding (**C**). In epithelial cells, this interaction destabilizes microvilli, thus contributing to attaching-effacing lesions (C1); in macrophages, this interaction inhibits phagocytic structure extension, thus leading to inhibition of phagocytosis (C2).

The epithelial cells of the human intestine exhibit a brush border of microvilli that protects the intestinal barrier from attacks by pathogens. However, some pathogens have developed strategies to overcome this physical protection. Thus, enteropathogenic *E. coli* (EPEC) is able to intimately bind to the apical plasma membrane of enterocytes and to trigger the disruption of microvilli and actin cytoskeleton organization underneath by creating lesions defined as attaching/effacing (A/E) lesions [58] (Figure 2). These lesions are the result of the action of proteins encoded by a pathogenicity island known as the locus of enterocyte effacement (LEE) [59]. LEE locus encodes both the intimin bacterial outer membrane protein, the bacterial receptor Tir and the EPEC type 3 secretion system (T3SS). Strikingly, the T3SS enables the bacteria to translocate into the host cell its own receptor, the Tir protein that is then exposed at the surface of the plasma membrane [60]. Interaction between intimine and Tir receptor triggers the signaling Nck/N-WASP/Arp2–3 pathway that activates actin polymerization and formation of pedestal-like structures that secure bacterial adherence by erasing microvilli from the apical surface (Figure 2). As well as injecting Tir inside the host cell, EPEC T3SS is responsible for the translocation of other bacterial effectors that are injected inside the target cell to hijack host cell functions and promote bacterial infection.

Among these, EspB, which is both a component of the T3SS machinery [61] and an effector detected inside the target cell cytoplasm, is essential for EPEC infection in humans [62]. After injection, EpsB interacts with α-catenin [63] and, most interestingly, with Myo1c, thus contributing to microvilli erasing as well as inhibiting phagocytosis [64,65]. Specifically, pull-down experiments with truncated constructs of myosin showed that EspB interacts with the C-terminal region of the motor domain of Myo 1c, thus suggesting that EspB competes with actin for Myo1c binding. Consistently, in epithelial Caco-2 cells infected by an *∆espB* mutant or an *espB∆mid* (deleted of the myosin interaction domain) mutant, the microvilli structure was not disrupted, as compared with the attaching/effacing (A/E) lesions induced by the WT strain infection. Thus, EspB and, specifically, its myosin-binding domain is necessary for effacing microvilli during EPEC infection [64] (Figure 2). In bone-marrow-derived macrophages of C57/BL6 mice, internalization of *∆espB* or *espB∆mid* mutants are 40% increased when compared to WT EPEC strain, thus suggesting that EspB and, more specifically, its myosin-binding domain are also required for inhibiting macrophages phagocytosis. Noteworthy, FLAG-tagged EspB interacts with many of the myosins involved in phagocytosis [65], such as Myo1a, II, V, VI and X [64]. Together, these data highlight that EPEC targets myosins, in particular Myo1c, to contribute to its adhesion to epithelial cells and to its evasion from macrophage phagocytosis.

### 3.2. Myosin II Contribution to N. gonorrhoeae Crossing over the Epithelial Barrier

*Neisseria gonorrhoeae*, a human obligatory pathogen, is able to infect the female reproductive tract and, more specifically, the endocervix, composed of columnar polarized epithelial cells. Endocervical cells form a series of junctions at their apical pole that stop pathogens from entering the paracellular space. Apical junctions are characterized by a ring of actomyosin cytoskeleton that, upon external signals, can regulate the epithelium permeability [66].

**Figure 3:**
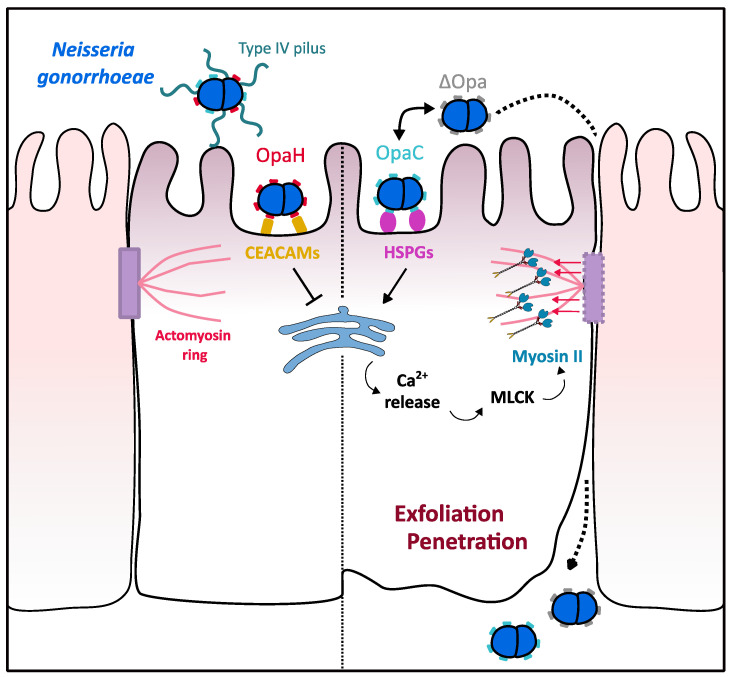
Role of myosin II in *N. gonorrhoeae* epithelial barrier crossing. *N. gonorrhoeae* is able to cross epithelial barriers by altering epithelium permeability. Interaction of the bacterial OpaC surface proteins with HSPGs triggers Ca^2+^ release from cell stocks, thus leading to the activation of myosin light chain kinase (MLCK) protein. Myosin II, thus activated by MLCK, generates strength on actin filaments associated with adherent junctions, which leads to exfoliation of epithelial cells and subsequent *Neisseria* crossing the endocervical epithelium. *Neisseria* is able to balance this mechanism by controlling through “phase variation” the expression of either OpaC that triggers exfoliation or OpaH that binds CEACAMs and ensure epithelial permeability.

*N. gonorrhoeae* has been shown to adhere to epithelial cells by its type IV pilus [66] and surface proteins, such as OpaH and OpaC that bind Carcinoembryogenic antigen-related cell adhesion molecule (CEACAMs) proteins and heparan sulfate proteoglycans (HSPGs), respectively [67,68]. After adhesion, the bacteria promote apical junction disassembly and subsequent exfoliation of epithelial cells, thereby increasing the permeability of the epithelium, which allows them to cross this physical barrier and invade the reproductive system. It has been shown that myosin II plays a role in mediating epithelial cell exfoliation, thus promoting *N. gonorrhoeae* infection [67]. Specifically, *N. gonorrhoeae* infection induces Ca^2+^ cytosolic release, as observed by the monitoring of two fluorescent Ca^2+^ level indicators, which in turn activates MLCK. MLCK is responsible for the increase in the phosphorylation level of myosin light chain (MLC), and therefore for the localization of myosin II at apical sites enriched in bacteria. This localization, observed by microscopy, could trigger endocytosis of E-cadherin from apical junctions, thus causing apical junction disassembly [68] (Figure 3). Noteworthy, the bacterial Opa proteins, specifically OpaH that binds CEACAMs proteins, are submitted to phase variation, i.e., a genetic control that induces ON/OFF expression [69], and therefore negatively regulates myosin II-mediated disassembly of apical junctions and reduce cell exfoliation. Together, these data show an indirect interplay between *N. gonorrhoeae* adhesion and myosin-II, which contributes to the colonization of endocervix tissue by bacteria.

### 3.3. Myosins I, II and VI Contribution to S. typhimurium Invasion and Replication in Epithelial Cells

*Salmonella typhimurium* is a facultative intracellular pathogen that invades its host epithelial cell in order to evade immune defenses and efficiently get nutrients to replicate. Crucial for cell invasion is the *S. typhimurium* Type 3 secretion system (T3SS) SPI-1 that injects into the host cell cytoplasm several effectors, among which SopE, SopE2 and SopB [70,71,72]. These effectors promote *Salmonella* invasion by the well-established Arp2/3-mediated-dependent membranes ruffles [71,73] and a more recently discovered process involving the myosin II-mediated invagination of *Salmonella* [73,74].

**Figure 4:**
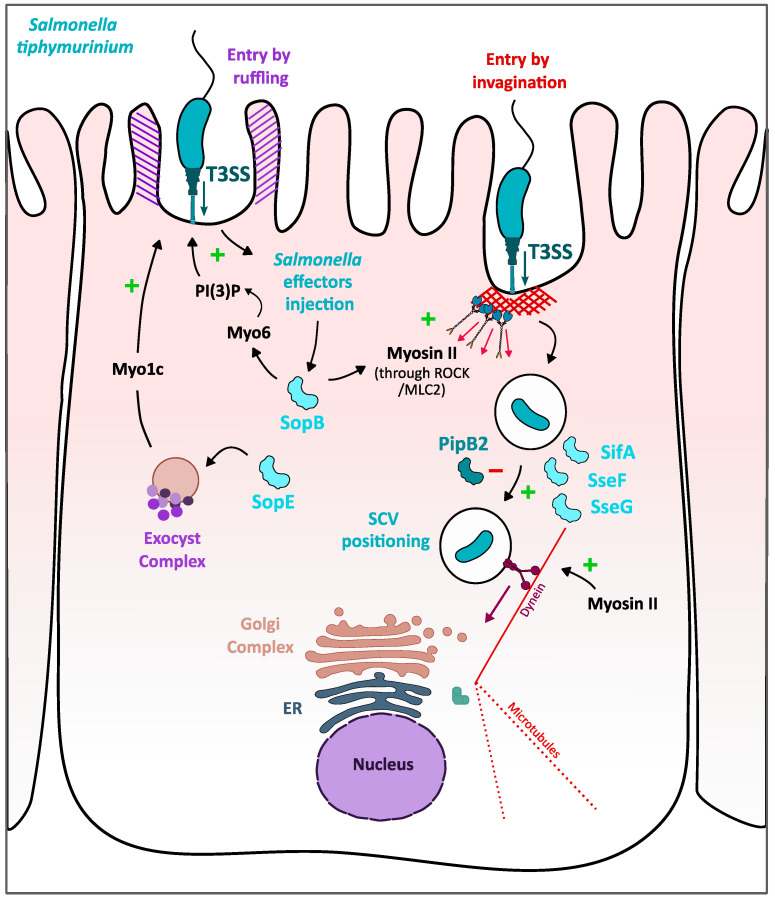
Role of myosins I, II and VI in *S. typhimurium* invasion and intravacuolar replication in epithelial cells. *S. typhimurium* enters the intestinal epithelial cells by triggering membrane ruffling (in purple strips) and invagination (in red strips). Both mechanisms are facilitated by myosins whose activity is induced by *Salmonella* injected effector SopB (light blue). In the “ruffling-dependent entry”, the role of myosin VI is indirect as it facilitates the recruitment of PI(3)P under the phagocytic cup, which in turn activates signaling pathways leading to recruitment of cytoskeletal proteins. Myo1c would also promote “ruffling-dependent entry” by recruiting additional membrane from the exocyst complex to elongate membrane ruffles. In the invagination process, the SopB/Rho-Rho-kinase/myosin II pathway generates force under the bacterial entry site hence pulling the bacterium inside the host. Then, *Salmonella* effectors, SseF, SceG and SifA, and myosin II are required for optimal positioning of the *Salmonella*-containing vacuole (SCV) to the perinuclear region for *Salmonella* replication.

The so-called “zip” model invasion process for *Salmonella* involves the translocation inside the host cell of SopE and SopE2 effectors, that both activate the Rho GTPases Rac1 or RhoG, thus activating the actin-nucleator complex Arp2/3 and finally promoting the formation of membrane ruffles by the reorganization of actin cytoskeleton under the site of bacterial adherence. This model has been recently completed by showing that (i) depletion of Myo6 reduces membrane ruffles under *Salmonella* invasion site and (ii) SopB, a *Salmonella* effector with phosphoinositide phosphatase activity [75] is able to recruit Myo6 at the invasion site, thus supporting a potential role of myosin VI in *Salmonella* invasion [76]. Consistently, Myo6, recruited to the plasma membrane at the *Salmonella* macropinocytic cup, was shown to facilitate invasion by triggering PI(3)P localization at the macropinocytic cup. PI(3)P localization to the invasion site would actually result from SopB-mediated dephosphorylation of plasma-membrane associated PI(3,4,5)P3 and PI(3,4)P2. The local concentration of PI(3)P mediated by the SopB/Myo6 pathway, in turn, activate PI3K activity, as Akt phosphorylation is reported to be dependent on Myo6. Therefore, Myo6 indirectly facilitates *Salmonella* invasion by promoting the recruitment of cytoskeleton proteins and the PI3K signaling in infected cells (Figure 4). *S. typhimurium* entry has been shown to benefit from another myosin, Myo1c, thanks to its role in recycling the lipidic rafts [18]. Massive recruitment of Myo1c at membrane ruffling sites and colocalization of the myosin with several actors of lipid raft were observed at the site of invasion: GPI lipid raft maker; RalA, a small cellular GTPase; and ExoC2, an exocyst complex component. RalA is required for the formation of *Salmonella* invasion foci [77] as once activated by the bacterial effector SopE. It activates the exocyst complex that brings additional membrane, thus participating in the extension of *Salmonella*-induced-membrane ruffles. This colocalization and the evidence that Myo1c knockdown in HeLa cells negatively impacts *S. typhimurium* invasion support that Myo1c participates in the process of *Salmonella* invasion by regulating the membrane ruffling or the transport of lipid raft coming from the exocyst complex (Figure 4).

Another pathway for the entry of *S. typhimurium* into the enterocytes is the myosin II-mediated invagination of the bacteria, an Arp2/3-independent entry mechanism involving both myosin II isoforms, NMIIA and NMIIB [74]. This mechanism would be mainly activated by *Salmonella* effector SopB, known to indirectly activate Rho GTPases such as Cdc42 and RhoG, and it is characterized by the presence of stress fibers-like structures attached to bacterial invasion site on one side and to cellular focal adhesions on the other, structures induced by the RhoA-mediated myosin II activation [78]. *Salmonella* uptake is significantly reduced when infected cells are treated with either the myosin II inhibitor blebbistatin, the inhibitor of Rho kinase Y27632 or the Rho protein inhibitor cell-permeable C3 transferase, thus proposing a link between *Salmonella* entry and Rho/myosin II-mediated contractility [74]. This pathway, when activated by SopB, create a contraction of stress-fibers like structures under the bacteria, thus inducing membrane invagination that can facilitate *Salmonella* Arp2/3-independent entry in its target cell (Figure 4).

Entry is not the only step of the *Salmonella* infectious cycle requiring myosins. Once internalized through Arp2/3-mediated membrane ruffles and/or myosin II-mediated stress fiber-like structures, *S. typhimurium* triggers the biogenesis of its replicative niche, an intracellular vacuole called *Salmonella*-containing vacuole (SCV). For *Salmonella* replication to start, the SCV needs to traffic and localize near the perinuclear region close to the Golgi apparatus. This positioning of the SCV is mediated by the second T3SS, SPI-2 that upon acidification of the vacuole injects inside the host cell, several effectors: SseF and SseG that recruit dynein, allowing transport of the SCV along microtubules to the nucleus [78,79]; PipB2 that promotes kinesin accumulation to the SCV for centrifugal extension of membranous *Salmonella*-induced filaments (Sifs) [80]; SifA that uncouple kinesin molecules from the SCV to prevent displacing SCV further from the nucleus [81]. At the perinuclear region, the SCV matures to avoid cellular degradation and intracellular bacterial replication starts [82] (Figure 4). Myosin II is required for the good positioning of the SCV near the nucleus, as myosin II inhibition by blebbistatin, and expression of a negative dominant of myosin II light chain that cannot be phosphorylated, leads to a peripherical dispersion of the SCV and inhibits intracellular growth of *S. typhimurium* [83]. As described for *Salmonella* entry, myosin II has been shown to be activated by SopB, through ROCK which is known to indirectly promote phosphorylation of myosin II light chain and, therefore, promotes myosin II activation [84]. Thus, myosin II could regulate the equilibrium between dynein and kinesis forces and counteract the PipB2 activity in order to prevent centrifugal displacement of the SCV. Hence, the two modes of *S. typhimurium* entry, namely “zip” invasion and invagination, but also the biogenesis of its replicative niche require indirect interactions between bacterial T3SS effectors and myosins, that involve well-known actin cytoskeleton controlling pathways.

### 3.4. Myosin IX and Myosins II/X Contribution to S. flexneri Epithelial Cell Invasion and Promotion of Cell-to-Cell Spreading

*Shigella flexneri*, as *S. typhimurium*, is a facultative intracellular bacterium that invades intestinal epithelial cells. Nonetheless, while *Salmonella* directly targets enterocytes, *S. flexneri* first crosses the intestinal epithelium barrier through the microfold “M” cells and is thus released on the other side, where it infects the enterocytes on their basal side [85]. To enter the host cell, *S. flexneri* injects through its T3SS, effectors that induce actin polymerization and the formation of filopods that engulf the bacteria [86,87,88]. After internalization, *S. flexneri* triggers the lysis of the vacuole, thus resulting in its release inside the host cell cytoplasm where it efficiently replicates [89] and polymerizes an actin tail at the bacterial pole that enables it to move inside the cell and to disseminate from cell-to-cell by inducing protrusion between cells [90].

**Figure 5:**
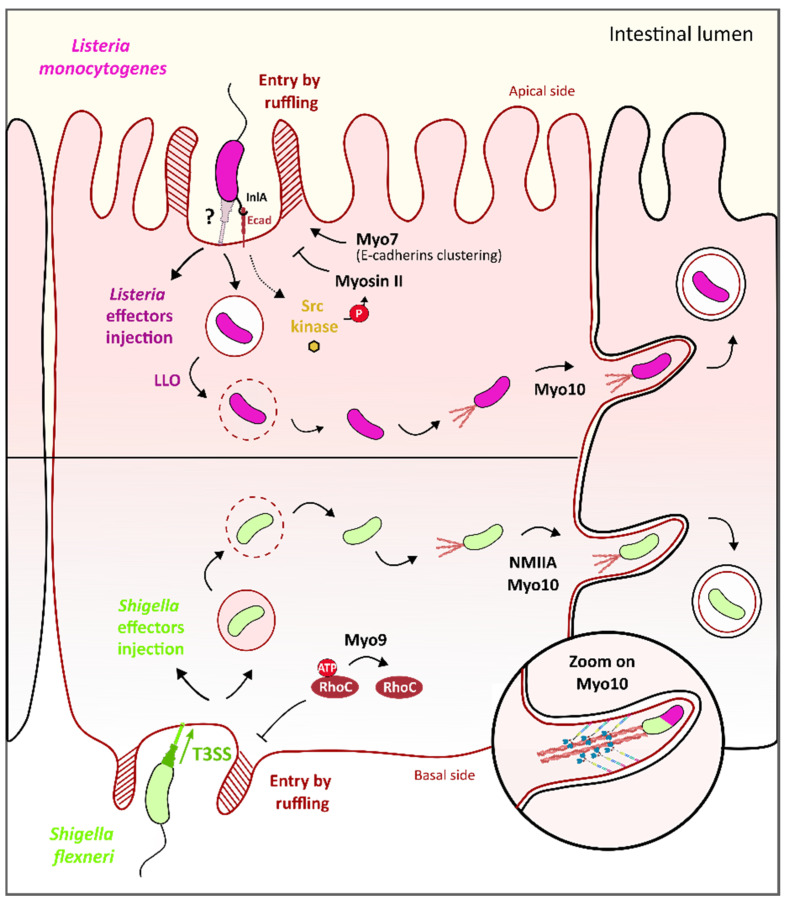
Role of Myosin IIA/VII/IX/X in *L. monocytogenes* and *S. flexneri* cell invasion and cell-to-cell spreading. *S. flexneri* (in light green, lower panel) entry on the basal side of epithelial cells by membrane ruffling is regulated by myosin IX. Specifically, Myo9b RhoGAP domain triggers the inactivation of RhoC, a Rho GTPase that activates actin polymerization, to stop protrusion formation/extension. Upon entering, *Shigella* is released into the cell cytosol and triggers the formation of an actin structure called “actin tail” at one pole to acquire actin-based intra- and intercell motility. Myosin X, as well as myosin IIa, promotes this step. Specifically, myosin X binds the membrane through its four-point-one, ezrin, radixin, moesin (FERM) domain and actin through its head domain. The motor domain activity is used as a strength to form the protrusion and “push” bacteria toward the neighboring cell. *L. monocytogenes* (in purple, upper panel) entry at the apical side of epithelial cells is induced by injection of bacterial effectors (in dark purple) inside the host cell cytoplasm that trigger actin polymerization and subsequent membrane ruffling. Entry of *L. monocytogenes* is facilitated by myosin VII/vezatin/cell adhesion complex pathway, which stabilizes membrane protrusions at the entry site of the bacteria. Conversely, membrane ruffling is balanced by myosin II to ensure the optimal enclosure of the bacteria inside the intracellular vacuole. Bacterial listeriolysin O (LLO) toxin lyses the vacuolar membrane, thus releasing *L. monocytogenes* in the cell cytosol. Here, bacteria promote the polymerization of actin at one of its poles, in an actin tail structure, which makes bacteria motile in order to escape cytosolic defenses such as autophagy and to spread to nearby cells. Similar to *S. flexneri*, myosin X plays a key role in this cell-to-cell spreading process.

The *S. flexneri* entry is dependent on indirect interactions with class IX myosin myr5. Myr5 and its human ortholog Myo9b localize at *Shigella* entry spots and accumulate in the membrane protrusions, where they are proposed to finely regulate the entry process of *S. flexneri.* First, in the presence of ezrin that binds actin to plasma membranes and localizes with myr5, the myosin head domain could facilitate the internalization of bacteria by stabilizing the newly polymerized *Shigella*-induced actin filaments [91]. Moreover, the myr5 GAP domain then inhibits the actin polymerization-associated Rho GTPases, particularly RhoC, to stop protrusion formation/extension [92] (Figure 5). This fine regulation by GTPases and myr5 would therefore regulate over time the polymerization of actin during the entry of the bacteria by preventing the formation of protrusions under invasive *Shigella*, which could repel it.

Once the bacteria enter the cell, they begin to replicate and to disseminate to other nearby cells through the apical junction complex (AJC) of the enterocytes. Myosin II and myosin X play an important role in pathogen-induced protrusion formation at the AJC. Specifically, drug inhibition of the myosin light chain kinase (MLCK), one regulator of myosin II results in less myosin-II and bacterial accumulation in cell–cell contact sites where none or few protrusions are formed [93]. Moreover, inhibition of NMIIA by siRNA reduces the formation of infection foci and results in a defect in bacteria-containing protrusions uptake by neighboring cells [94] (Figure 5). Myosin X was also found in membrane projections associated with bacteria and, more precisely, localized all along *Shigella* bacteria; interestingly, its knockdown by siRNA results in a 25% reduction of membrane protrusions, an enlargement at the base of the protrusion and a significant decrease in dissemination ability of the bacteria [95]. It was shown that the Myo10 head and neck domains were sufficient for Myo10 localization alongside the bacteria while the tail region PH domain alone is responsible for the binding of Myo10 to the plasma membrane in protrusions, as expected regarding the property of this domain to bind phosphoinositides [95] (Figure 5, zoom). Thus, the PH domain was proposed to link the Myo10 to the plasma membrane of the *Shigella*-induced protrusions and the motor domain responsible for the forward movement of Myo10 on actin filaments toward the tips of the protrusions, which could facilitate the extension of *Shigella*-induced protrusions to the nearby cells. Although the molecular mechanisms induced by the bacteria are still unclear, these data reveal that Myosin II and myosin X contribute to Shigella-induced protrusion formation, and consequently, to its dissemination in the intestinal epithelium.

### 3.5. Myosin VII and Myosins IIA/X Contribution to L. monocytogenes Epithelial Cell Invasion and Promotion of Cell-to-Cell Spreading

*Listeria monocytogenes* belongs to foodborne bacteria such as *S. typhimurium* or *S. flexneri* that invade epithelial host cells by triggering their entry inside these cells. The entry of *L. monocytogenes* is mediated by two surface proteins called internalins A and B (InlA and InlB) that bind cell receptors and subsequently trigger actin polymerization and cell membrane remodeling [96]. While InlB interacts with the Met receptor to trigger *Listeria* entry, InlA specifically interacts with the E-cadherin protein, thus leading to the activation of Src kinase and cognate tyrosine phosphorylation, and finally, allows cytoskeleton rearrangement through the recruitment of the actin nucleator complex Arp2/3 [97,98]. However, another cytoskeleton-associated protein, namely Myo7a and its interaction with vezatin, would also be involved in this process, as inhibition of Myo7a with BDM leads to the defect of entry of the bacteria. The Myo7a tail domain is required for the recruitment of Myo7a at the internalization site, and plus-end motor activity of Myo7a contributes to *Listeria* entry, specifically in steps that follow the early clustering of *Listeria* engaged E-cadherin receptors on the plasma membrane [99] (Figure 5). Moreover, NMIIA has been depicted as a regulator of *Listeria* entry inside HeLa cells as the Src kinase triggers the phosphorylation of NMIIA heavy chain on Tyr158 that results in fewer bacteria uptake [100] (Figure 5). Thus, indirect interactions with Myo7a and NMIIA finely control the invasion of epithelial cells by *Listeria.*

Once the bacteria enter the cell, *L. monocytogenes* injects listeriolysin O (LLO), a pore-forming protein that promotes vacuole rupture and bacteria escapes into the cell cytoplasm. In the cytoplasm, *L. monocytogenes* uses actin-based motility to move inside the host cell cytoplasm and propel bacteria through membrane protrusions into neighboring cells, thereby efficiently disseminating in the epithelium. As previously described for Shigella, Myo10, thanks to its motor domain, significantly contribute to the formation and extension of cell protrusions induced by *Listeria* infection and, consequently, for *Listeria* cell-to-cell spreading [95] (Figure 5).

### 3.6. Myosin II Contribution to C. trachomatis Exit out of the Host Cell

**Figure 6:**
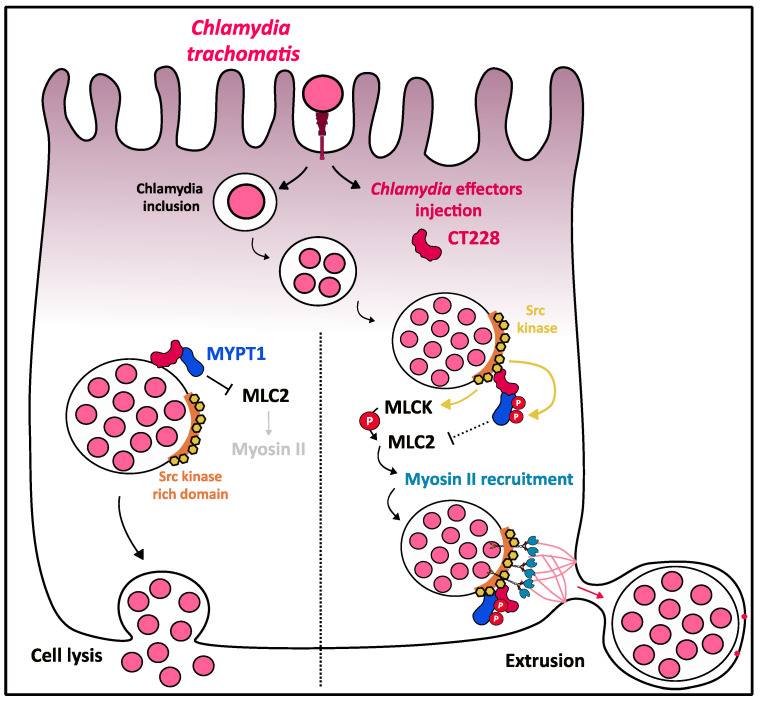
Role of myosin II in C. trachomatis cellular exit by extrusion. After internalization, *C. trachomatis* efficiently replicates inside a specialized vacuole called inclusion. On the inclusion membrane, *Chlamydia* effector CT228 is found to colocalize with MYPT1 that is recruited early during infection. The localization and activation status of MYPT1 regulates the cell exit strategy of the bacteria. MYPT1 activated state (non-phosphorylated) is associated with *Chlamydia* exit through lysis. Extrusion pathway is associated with relocalization of MYPT1 in Src kinase-rich regions on the inclusion membrane in which MYPT1 could progressively be phosphorylated on both inhibitory sites, T696 and T853 by Src kinase and therefore inactivated. This may lead to the MLC2 release from its interaction with MYPT1 and its subsequent availability to be phosphorylated and activated by MLCK, triggering the recruitment of myosin II on Src kinase-rich regions. Myosin II is then used as a tether to walk along the actin to the plasma membrane and create an extrusion.

Another intracellular pathogenic bacterium, *Chlamydia trachomatis*, targets the host cytoskeleton and, more specifically, myosin II, for the final step of its infectious cycle, i.e., the exit out of the cell. *C. trachomatis* enters epithelial cells thanks to T3SS-mediated injection of effectors that induce actin remodeling [101] and subsequent internalization inside an intracellular compartment called inclusion [102]. Inclusion membrane is enriched in *Chlamydia* proteins called Incs that, once injected inside the host cell, interact with host factors, thus making the inclusion an efficient replicative niche for the bacteria [103,104]. After bacteria replication, *C. trachomatis* infectious cycle ends with the exit of the cell, either by lysis of the host cell or by an active mechanism known as extrusion, which relies on myosin II (Figure 6).

In the late stages of infection, inclusions are characterized by transient septation and a peripherical location accompanied by outward budding, described as the extrusion exit mechanism. Actin polymerization and myosin-II are required for extrusion formation, as latrunculin B and blebbistatin-treated HeLa infected cells showed inhibition of extrusion formation. Inhibition of Rho GTPase also results in the arrest of extrusion formation at the “pinching” step [103]. Importantly, actin is recruited at the cytosolic face of inclusion membranes in *Chlamydia*-infected HeLa cells by a process involving septins, myosin II and Src-family kinase (SRK) on the host side and the T3SS-mediated injection of bacterial effectors on the other side [104]. Specifically, the C-terminal domain of the *C. trachomatis* Inc protein CT228 interacts with the leucine-zipper domain of the myosin phosphatase target subunit 1 MYPT1, resulting in colocalization of CT228 and MYPT1 at the inclusion membrane [105]. MYPT1 is recruited earlier during the infection, but MYPT1 appears progressively relocated in an inactive T696- and T853-phosphorylated state at inclusion membrane microdomains enriched with Src-family kinase (Figure 6). MYPT1 phosphorylation was shown to inhibit its phosphate activity, thus leading to the loss of interaction with its target myosin light chain 2 (MLC2) [106], and consequently to the activation of myosin II motor domain and the formation of extrusion to promote the exit of *Chlamydia* (Figure 6). As MLCK is known as a positive regulator of MLC2, an antagonistic role of MLCK/MYPT on the activation state of MLC2 could be suggested to control the balance between extrusion signals (generated by active-MLC2) and lysis signals (generated by inactive-MLC2) [107]. Thus, the MYPT1/MLC2/myosin II pathway finely regulates the balance between the two modes of *Chlamydia* exit from the cell.

## 4. Discussion and Conclusions

Myosins are a crucial and very conserved element of the cytoskeleton that plays a key role in the homeostasis and general biology of the eukaryotic cells, including cell migration and adhesion, signal transduction, intracellular and membrane trafficking, involved in particular in endocytosis, phagocytosis and vesicular trafficking. The role of myosins in essential cell processes makes them ideal targets for bacteria to hijack host cell pathways to their benefit and to evade immune defenses in order to efficiently infect, replicate and disseminate in their host. Thus, some extracellular bacteria, but even more intracellular bacteria have evolved sophisticated strategies that target myosins to go through different stages of their infectious cycle: (i) adhesion to the host cell, for EPEC; (ii) invasion, i.e., entry into non-phagocytic cells, for *Salmonella*, *Shigella*, and *Listeria*; (iii) evasion from the cytosolic autonomous cell defenses among which autophagy, for *Shigella* and *Listeria*; (iv) biogenesis of a replicative niche, such as the vacuole of *Salmonella*; (v) dissemination in tissues by the cell-to-cell spreading for *Shigella* and *Listeria*; (vi) exit out of the host cell for *Chlamydia*; (vii) evasion from phagocytosis for EPEC. Noteworthy, bacteria can activate or inhibit the myosin-dependent processes, depending on the infection step involved. Importantly, various myosins are targeted by bacteria, but the prevalent involvement of myosin II in the host–bacteria relationship can be noted, possibly due to its key role in many mechanical tasks, including the regulation of adhesion and intracellular trafficking (endocytosis, exocytosis, phagocytose and vesicular transport). With the exception of the bacterial effector EspB from EPEC, which directly interacts with the C-terminal domain of myosin Ic, thus competing with actin for binding this myosin, most of the mechanisms involved in the manipulation of myosins are based on indirect interactions leading to post-translational modifications of myosins or triggering complex signaling pathways that activate or inhibit myosins.

Myosins are not the only cytoskeletal protein targeted by a wide variety of bacteria. Indeed, the actin cytoskeleton (-F, -G and polymerization) seems to be a privileged target for bacteria [108], particularly with regard to bacterial entry [109,110,111] or propagation between neighboring cells [112,113]. Interestingly, the study of the interaction between bacteria and the host cell actin cytoskeleton has advanced not only our knowledge in bacteriology but also our understanding of the dynamic mechanisms governing the host cell cytoskeleton. Thus, basic research on *Listeria* infectious cycle, and in particular its entry into epithelial cells, its intracellular motility and its cell-to-cell dissemination has allowed the astonishing discovery of the first nucleation promoting factor (NPF), ActA, a *Listeria* protein surface that allowed the recruitment of cellular protein vasodilator-stimulated-phosphoprotein (VASP) [114,115] and consequently actin polymerization at one pole of the bacteria. This breakthrough discovery has paved the way for the discovery of other NPFs and the understanding of molecular mechanisms that finely control the dynamics of actin polymerization. Similarly, it can be expected that the deepening of our knowledge of the host–pathogen interaction could lead to the discovery of new mechanisms associated with myosins.
